# A Stochastic FE^2^ Data-Driven Method for Nonlinear Multiscale Modeling

**DOI:** 10.3390/ma14112875

**Published:** 2021-05-27

**Authors:** Xiaoxin Lu, Julien Yvonnet, Leonidas Papadopoulos, Ioannis Kalogeris, Vissarion Papadopoulos

**Affiliations:** 1 Shenzhen Institute of advanced electronic materials, Shenzhen Institutes of Advanced Technology, Chinese Academy of Sciences, Shenzhen 518103, China; luxiaoxin.cassie@gmail.com; 2 MSME, University Gustave Eiffel, CNRS UMR 8208, F-77454 Marne-la-Vallée, France; 3 Department of Civil Engineering, National Technical University of Athens, 15780 Athens, Greece; lew.papado@hotmail.com (L.P.); yianniskalogeris@gmail.com (I.K.); vissarion.papadopoulos@gmail.com (V.P.)

**Keywords:** data-driven, multiscale, nonlinear, stochastics, neural networks

## Abstract

A stochastic data-driven multilevel finite-element (FE${}^{2}$) method is introduced for random nonlinear multiscale calculations. A hybrid neural-network–interpolation (NN–I) scheme is proposed to construct a surrogate model of the macroscopic nonlinear constitutive law from representative-volume-element calculations, whose results are used as input data. Then, a FE${}^{2}$ method replacing the nonlinear multiscale calculations by the NN–I is developed. The NN–I scheme improved the accuracy of the neural-network surrogate model when insufficient data were available. Due to the achieved reduction in computational time, which was several orders of magnitude less than that to direct FE${}^{2}$, the use of such a machine-learning method is demonstrated for performing Monte Carlo simulations in nonlinear heterogeneous structures and propagating uncertainties in this context, and the identification of probabilistic models at the macroscale on some quantities of interest. Applications to nonlinear electric conduction in graphene–polymer composites are presented.

## 1. Introduction

Predicting the nonlinear behavior of materials from knowledge of their microstructure is a critical topic in engineering. For example, the development of 3D-printed micromaterials [1,2,3] or of nanomaterials [4,5] with nonlinear behaviors opens exciting opportunities for designing innovative functionalized and enhanced engineering systems. While linear effective properties of heterogeneous materials can be accurately estimated though either analytical [6,7] or numerical techniques [8], predicting the effective behavior of nonlinear materials requires more advanced techniques.

A direct but limited approach is the use of the representative volume element (RVE) to calibrate an empirical nonlinear model. A limitation of such techniques is the number of parameters to be calibrated for complex, nonlinear, or multiphysics problems. To more accurately describe the behavior of general nonlinear materials, the so-called multilevel finite-element (FE${}^{2}$) method [9,10,11,12,13,14,15,16] or computational homogenization has been developed in recent years. In this approach, an RVE is associated to each Gaussian point of a finite-element macrostructure, and a nonlinear problem must be solved in each integration point and for each iteration of the macrosolving procedure. The drawback of this method, however, is that it induces unaffordable computational times in practical applications.

Several strategies were developed recently to alleviate FE${}^{2}$ calculations. First, the strategy relies on reducing micro-RVE computations through efficient techniques such as model-order reduction [17,18], fast Fourier transform [19,20], wavelet transforms [21], NTFA [22], self-clustering analysis (SCA) [23,24], or GPU acceleration [25]. In [26], He et al. developed an adaptive strategy to reduce microcalculations by constructing the reduced basis on the fly during the macroscale calculation.

Another idea, initiated in [27,28], is the use of so-called data-driven approaches in which microscale calculations are performed offline, and are then used as data in an online stage to reconstruct the macroscopic (effective) behavior. For this purpose, several techniques were proposed, including interpolation methods [27,29], neural networks [28,30,31,32,33,34,35], Bayesian inference [36], Fourier series expansions [37], or Gaussian process regression [38]. In the related techniques, offline data collection is used in a regression process to construct an accurate surrogate model whose evaluation is several orders of magnitude lower than that performing one RVE nonlinear calculation. A critical comparison of several regression techniques used in data-driven multiscale approaches can be found in [39]. In [40], Avery et al. investigated and discussed several regression methods with ANN in homogenization problems of hyperelastic woven composites, and demonstrate its use in advanced dynamic or fluid structure applications. Recent advances of data-driven techniques, including handling history-dependent behaviors such as plasticity, can be found in [35,41,42]. On-the-fly construction of the surrogate model by probabilistic machine learning was proposed in [38]. Developments of neural-network techniques in FE${}^{2}$, including feed-forward and recurrent neural networks, can be found in [31,41]. In [43,44], a manifold-based nonlinear reduced-order model in tandem with a digital database was developed for the nonlinear multiscale analysis of hyperelastic structures involving neural networks, a kernel inverse/reconstruction map, and dimension reduction through an isomap.

Stochastic extensions of data-driven methods in multiscale applications are relatively new and unexplored. One of the first analyses in this context can be found in [45,46], where the NEXP method [27] was extended to stochastic problems. In these studies, stochastic parameters were introduced within the surrogate model using a separated representation-interpolation technique. Probability density functions related to the nonlinear macroscale problem were identified. In [47], a machine-learning strategy based on a three-dimensional convolutional neural network was introduced to evaluate the linear effective properties of random materials from geometrical descriptions of RVE. In [24], a framework for uncertainty quantification in a data-driven approach was proposed where self-consistent clustering analysis (SCA) [23,24] was used to reduce computational times in the learning step.

In this paper, the use of data-driven methods for heterogeneous nonlinear materials with uncertainties at both the micro- and the macroscale is addressed. Taking into account uncertainties in nonlinear multiscale methods implies (a) constructing a probabilistic surrogate macromodel from microcalculations, allowing for generating realizations of the macroresponse for a given macroloading; and (b) performing Monte Carlo simulations of the model at the macroscale to quantify uncertainties on the quantities of interest in the structure. In view of its immense computational requirements, direct use of FE${}^{2}$ for stochastic nonlinear two-scale analysis is not possible. However, data-driven FE${}^{2}$ approaches have comparable computational costs as compared to classical (one-scale) FEM calculations, and they open the route to developing stochastic two-scale nonlinear approaches. To the best of our knowledge, this problem remains relatively unexplored in the literature. A new stochastic data-driven approach based on RVE calculations was developed for taking into account random effects in nonlinear heterogeneous structures. First, preliminary RVE calculations were performed. These calculations include several microstructural features that varied, such as the distribution of heterogeneities and its volume fraction. Then, for each realization of the random microstructure, the space of macroscopic loading was sampled, and boundary conditions were prescribed on the RVE. Subsequently, the nonlinear problem was solved by FEM. This large database was used to construct a surrogate model whose inputs were the macroloading and the volume fraction, and its output was the macroscopic (homogenized) response. A new hybrid neural-network–interpolation (NN–I) surrogate model is proposed to provide an accurate response with a limited number of realizations. Once constructed, this model can be used within stochastic analysis of two-scale nonlinear structure calculations. At the macroscale, the volume fraction of heterogeneities is considered to be random here, and it was modeled as a stochastic field with given probabilistic characteristics. Then, during the macro-non-linear resolution, solving the full nonlinear RVE was replaced by the proposed fast surrogate model, which allowed for performing hundreds of macro-non-linear calculations at the cost of classical FEM problems. As a result, statistical postprocessing can be performed on the macroquantities of interest, and probabilistic models could be identified.

The novelties of this paper are twofold. The first is the proposed neural-network–Interpolation FE${}^{2}$ method, which is an extension of our previous neural-network FE2 method, developed in [28,30]. The NN–I scheme allows for modeling the stochastic spatial variability of the volume fraction in the frame of the FE${}^{2}$ procedure, leading to the improved accuracy of the surrogate model when limited data are available. The second novelty is the application of this machine-learning method to nonlinear multiscale stochastic problems. Using the proposed approach, FE${}^{2}$ calculations can be reduced by several orders of magnitude, allowing for Monte Carlo simulation on stochastic nonlinear multiscale structures. It is demonstrated for the first time that uncertainties can be propagated in this context, and probabilistic models can be identified.

The paper is organized as follows. Section 3 presents the equations of the nonlinear RVE problem, and the definitions of the input (macroelectric load) and output (homogenized electric flux) in the nonlinear composite. Section 4 introduces the hybrid neural network/interpolation scheme, and its construction using offline data on RVE is described. In Section 5, the present stochastic data-driven strategy is proposed. Lastly, numerical examples are presented in Section 6.

## 2. Brief Review of FE${}^{2}$ Method for Nonlinear Conduction

**Figure 1:**
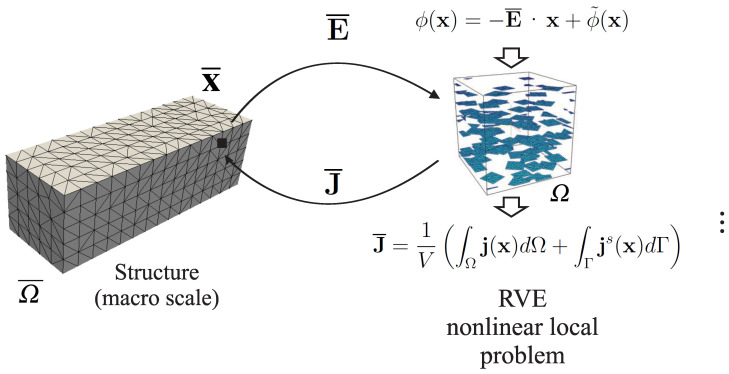
Schematic of classical FE${}^{2}$ method for nonlinear heterogeneous conduction problem (adapted from [8]).

The multilevel finite-element method [9,10], also called FE${}^{2}$ in the literature, as it involves two levels of finite-element simulations, and independently proposed by several other authors and groups [11,12,13,14,15,16], was introduced as a general multiscale method for solving nonlinear heterogeneous structural problems. The basic underlying idea is that two levels of finite elements must be concurrently solved, one for each scale. At the macroscale, each integration point of the finite-element mesh is associated with a representative volume element (RVE). Boundary conditions depending on the macroscopic state (strain, electric field, etc.) are prescribed on the boundary of each RVE. After solving each nonlinear problem at each integration point, the appropriate macroscopic response (stress, electric flux), is averaged over the RVE and provided at the macrointegration point. Then, the macroscopic constitutive law is available only through solving a nonlinear problem. These operations are repeated until convergence is reached at both scales (see Figure 1).

For the sake of simplicity, a brief review of the method in a context of nonlinear conduction is presented. We consider a macroscopic structure associated with a domain $\bar{\Omega }\subset R^{3}$, with a boundary $\partial \bar{\Omega }$. The assumption of scale separation is adopted (an extension of the method to second-order homogenization can be found in [14]). The microstructure was assumed to be characterized by an RVE associated with a domain $\Omega \subset R^{3}$, with boundary ∂Ω.

In the context of nonlinear electric conduction, electric field E(x) is related to the electric flux, or electric displacement j(x) by a nonlinear local constitutive relationship. Electric field E is defined by E(x)=−∇ϕ(x), where ϕ is the electric potential, ∇(.) is the gradient operator, and x is a material point within Ω. In the following, $\bar{\left(.\right)}$ notations denote macroscale quantities. For a given macroscopic electric field $\bar{E}$, the RVE problem is to find ϕ(x), such that

(1) $$\begin{array}{c} \nabla \cdot j(x)=0\;\forall x\in \Omega , \end{array}$$

where ∇·(.) is the divergence operator. The constitutive law is given by

(2) $$\begin{array}{c} j\left(x\right)=F^{nl}\left(E(x)\right). \end{array}$$

where $F^{nl}$ is a local nonlinear operator (specified in Section 3). The equilibrated electric field should satisfy

(3) $$\bar{E}=\frac{1}{V}\int_{\Omega }E\left(x\right)d\Omega ,$$

where *V* is the volume of Ω. Equation (3) can be verified, e.g., by the following boundary condition:

(4) $$\phi \left(x\right)=-\bar{E}\cdot x+\tilde{\phi }\left(x\right)\;\;\mathrm{on}\;\partial \Omega ,$$

where $\tilde{\phi }\left(x\right)$ is a periodic function over Ω.

In the presence of imperfect interfaces and surface electric flux along interfaces (see [48]), the effective electric current $\bar{J}$ is defined according to

(5) $$\bar{J}=\frac{1}{V}\left(\int_{\Omega }j\left(x\right)d\Omega +\int_{\Gamma }j^{s}\left(x\right)d\Gamma \right),$$

where $j^{s}$ is a surface electric flux (see Section 3). In the so-called FE${}^{2}$ method, the constitutive law $\bar{J}$ - $\bar{E}$ is unknown, but can be numerically obtained by solving a nonlinear problem over the RVE, detailed as follows (see Figure 1):

Given $\bar{E}$:

1. Prescribe boundary conditions (4) on ∂Ω.
2. Use a numerical method such as FEM with an iterative solver such as the Newton method to solve nonlinear Problems (1), (2), and (4) (see details in the following).
3. Compute the spatial average of the electric flux over the RVE to obtain $\bar{J}$.

In what follows, a detailed numerical implementation of a FE${}^{2}$ problem in a context of nonlinear electric conduction is presented to better understand where Problems (1), (2), and (4) must be solved within finite-element calculation at the macroscopic scale. The macroscopic problem at the macroscale is given by

(6) $$\nabla \cdot \bar{J}=0\;\;\mathrm{in}\;\bar{\Omega },$$

with boundary conditions:

(7) $$\bar{\phi }={\bar{\phi }}^{*}\;\;\mathrm{on}\;\partial {\bar{\Omega }}_{\phi },\;\;\bar{J}\cdot n={\bar{J}}_{n}^{*}\;\;\mathrm{on}\;\partial {\bar{\Omega }}_{J},$$

where and ${\bar{\Omega }}_{\phi }$ and $\partial {\bar{\Omega }}_{J}$ denote the Dirichlet and Neumann complementary boundaries, respectively.

In what follows, we assume ${\bar{J}}_{n}^{*}=0$. Then, the weak form corresponding to (6) is given by:

(8) $$\int_{\bar{\Omega }}\bar{J}\left(\bar{\phi }\right)\cdot \nabla \left(\delta \bar{\phi }\right)d\Omega =\bar{R}\left(\bar{\phi }\right)=0.$$

Problem (8) is nonlinear. Then, a Newton method is employed to solve it. A first-order Taylor expansion of $\bar{R}\left(\bar{\phi }\right)$ gives

(9) $$\bar{R}\left({\bar{\phi }}^{k}+\Delta \bar{\phi }\right)≃\bar{R}\left({\bar{\phi }}^{k}\right)+D_{\Delta \bar{\phi }}\bar{R}\left({\bar{\phi }}^{k}\right),$$

where ${\bar{\phi }}^{k}$ is a solution provided at a previous iteration, and $D_{\Delta \bar{\phi }}\bar{R}\left(\bar{\phi }\right)$ is the Gateaux derivative, expressed by

(10) $$D_{\Delta \bar{\phi }}\bar{R}\left({\bar{\phi }}^{k}\right)={\left[\frac{d}{d\alpha }\left(\bar{R}\left(\bar{\phi }+\alpha \Delta \bar{\phi }\right)\right)\right]}_{\alpha =0}.$$

The corresponding linearized problem is given by

(11) $$D_{\Delta \bar{\phi }}\bar{R}\left({\bar{\phi }}^{k}\right)=-\bar{R}\left({\bar{\phi }}^{k}\right),$$

with

(12) $$D_{\Delta \bar{\phi }}\bar{R}\left({\bar{\phi }}^{k}\right)=-\int_{\bar{\Omega }}\bar{k}\left(\phi^{k}\right)\nabla \left(\Delta \bar{\phi }\right)\cdot \nabla \left(\delta \phi \right)d\Omega .$$

More details can be found in [48]. Classical FEM discretizing of (11) leads to linear system

(13) $${\bar{K}}_{T}\left({\bar{\phi }}^{k}\right)\Delta \bar{\phi }=-R\left({\bar{\phi }}^{k}\right).$$

Then, the macroelectric potential is updated according to

(14) $${\bar{\phi }}^{k+1}={\bar{\phi }}^{k}+\Delta \bar{\phi }$$

and (13) is solved until a convergence criterion is reached. In FE${}^{2}$, the main source of computational cost is the numerical evaluation of $\bar{J}$ and $\bar{k}$, obtained by solving nonlinear RVE Problems (1), (2), and (4) at each Gaussian point. To address this issue, we introduce a data-driven approach where the estimation of $\bar{J}$ is provided by a neural-network-based surrogate model. Tangent matrix $\bar{k}$ can be computed by a perturbation approach as

(15) $${\left({\bar{k}}_{T}\right)}_{ij}\left(\bar{E}\right)=\frac{\partial \bar{J}}{\partial \bar{E}}≃\frac{{\bar{J}}_{i}\left(\bar{E}+\delta {\bar{E}}^{\left(j\right)}\right)-{\bar{J}}_{i}\left(\bar{E}\right)}{\alpha }$$

with

(16) $$\delta {\bar{E}}^{\left(j\right)}=\alpha e_{j},$$

where $e_{j}$ is a unitary vector basis, and α<<1 a perturbation parameter.

Then, to compute macro-FEM nonlinear calculations, relationship $\bar{J}={\bar{F}}^{nl}\left(\bar{E}\right)$ is missing. In [30], we proposed a surrogate model to construct such a relationship using neural networks. In the present paper, this idea is extended to random microstructures, as detailed in the next section.

## 3. Micro-Non-Linear Conduction Model for Graphene-Reinforced Composites

**Figure 2:**
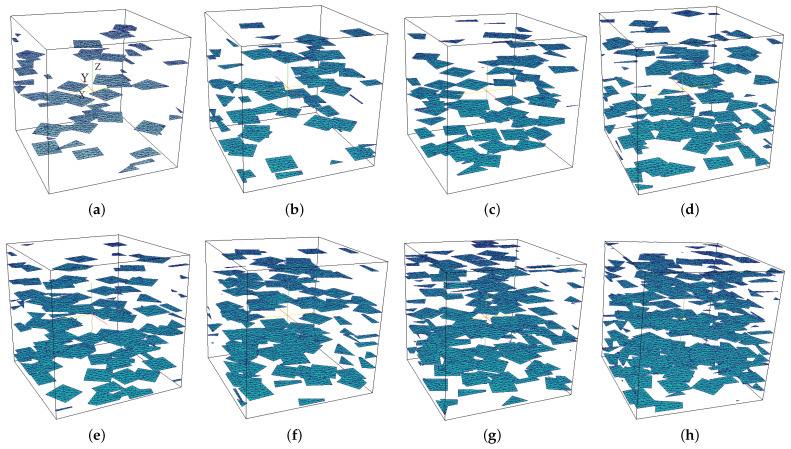
Realizations of microscopic RVE with various graphene volume fractions: (**a**) 0.53 vol%, (**b**) 0.66 vol%, (**c**) 0.79 vol%, (**d**) 0.92 vol%, (**e**) 1.05 vol%, (**f**) 1.19 vol%, (**g**) 1.32 vol%, (**h**) 1.58 vol% [30].

In this section, the nonlinear conduction model in graphene-reinforced polymer composites is defined. The nonlinear RVE problem is described as follows. The microstructure was assumed to be characterized by an RVE defined in a domain $\Omega \subset R^{3}$ that contained *N* randomly distributed planar multilayer graphene sheets (see Figure 2). The graphene sheets were assumed to be aligned along the x−y plane. We chose this configuration for two reasons: (i) when samples made of graphene-reinforced polymer are obtained via injection molding, the graphene sheets can be aligned in the direction of the polymer flow [49]. Then, this configuration is representative of samples manufactured by the injection-molding process. Second, such an orientation induces strong anisotropy of the effective nonlinear conductive behavior of the material. The potential of the present approach to deal with such a challenging problem is then illustrated.

To avoid meshing their thickness [48], the graphene sheets were modeled as highly conducting imperfect surfaces here [50]. The graphene surfaces with zero thickness are collectively denoted by Γ. The nonlinear behavior is related to the electric tunnelling effect here, which may be an explanation for the observed nonlinear behavior and low percolation thresholds in the nanocomposites (see [30,48]).

The energy of the system is defined by

(17) $$\begin{array}{c} W=\int_{\Omega }\omega^{b}\left(x\right)d\Omega +\int_{\Gamma }\omega^{s}\left(x\right)d\Gamma , \end{array}$$

where density functions $\omega^{b}$ and $\omega^{s}$ are the bulk and surface density functions, respectively, expressed by

(18) $$\begin{array}{c} \omega^{b}\left(x\right)=\frac{1}{2}j\left(x\right)\cdot E\left(x\right),\;\;\;\omega^{s}\left(x\right)=\frac{1}{2}j^{s}\left(x\right)\cdot E^{s}\left(x\right). \end{array}$$

In (18), $E^{s}\left(x\right)$ and $j^{s}\left(x\right)$ are the surface electric field and surface current density with respect to the graphene sheets, where $E^{s}=PE=-P\nabla \phi$, where P=I−n⊗n is a projector operator characterizing the projection of a vector along the tangent plane to Γ at a point x∈Γ, and n is the unit normal vector to Γ.

The nonlinear electric-conduction law including the tunneling effect in the polymer matrix is given by

(19) $$j=\left\{\begin{array}{cc} k_{0}^{p}E & \mathrm{if}\;d\left(x\right)\leq d_{cut},\\ G\left(E,d\right)\frac{E}{\left|E\right|} & \mathrm{if}\;d\left(x\right)>d_{cut}, \end{array}\right.$$

where $d_{cut}$ is a cut-off parameter, and $k_{0}^{p}$ is the electric-conductivity tensor of the polymer matrix without tunneling effects. The distance function between graphene sheets d(x) is defined here as the sum of the two smallest distances between a point x in the polymer matrix and the two nearest-neighbor graphene sheets (see more details in [48]). Nonlinear tunneling current G was proposed by Simmons [51] as

(20) $$\begin{array}{cc} G\left(E,d\right) & =\frac{2.2e^{3}E^{2}}{8\pi h_{p}\Phi_{0}}\exp\left(-\frac{8\pi }{2.96h_{p}eE}{\left(2m\right)}^{\frac{1}{2}}\Phi_{0}^{\frac{3}{2}}\right)\\ \; & \;+\left[3\cdot \frac{{\left(2m\Phi_{0}\right)}^{\frac{1}{2}}}{2d}\right]{\left(e/h_{p}\right)}^{2}Ed\exp\left[-\left(\frac{4\pi d}{h_{p}}\right){\left(2m\Phi_{0}\right)}^{\frac{1}{2}}\right]. \end{array}$$

Above, $\Phi_{0}$ and *d* denote barrier height and barrier width, respectively, *e* and *m* are the charge and the effective mass of electron, respectively, and $h_{p}$ is the Planck constant. Surface current density $j^{s}$ of graphene surface Γ is related to surface electric field $E^{s}$ [50] through

(21) $$j^{s}\left(x\right)=k^{s}E^{s},$$

where $k^{s}$ denotes the the surface conductivity of the graphene. This tensor can be related to the conductivity of the volume (multilayer) graphene as:

(22) $$k^{s}=hS^{*},\;\;\;S^{*}=k^{g}-\frac{\left(k^{g}n\right)⊗\left(k^{g}n\right)}{k^{g}:\left(n⊗n\right)}.$$

where *h* is the thickness of the graphene sheet.

Considering the constitutive equations above, and minimizing the electric power (17) with respect to the electric potential field, the weak form is obtained as

(23) $$\begin{array}{c} \int_{\Omega }j\left(\phi \right)\cdot \nabla \left(\delta \phi \right)d\Omega -\int_{\Gamma }P\nabla \phi \cdot k^{s}P\nabla \left(\delta \phi \right)d\Gamma =0, \end{array}$$

where $\phi \in H^{1}\left(\Omega \right)$, ϕ satisfying the Dirichlet boundary conditions over ∂Ω and $\delta \phi \in H^{1}\left(\Omega \right)$, δϕ=0 over ∂Ω. The RVE is subjected to boundary conditions (4). The weak form (23) can be solved by the finite-element method.

## 4. Stochastic Nonlinear Machine-Learning Model

**Figure 3:**
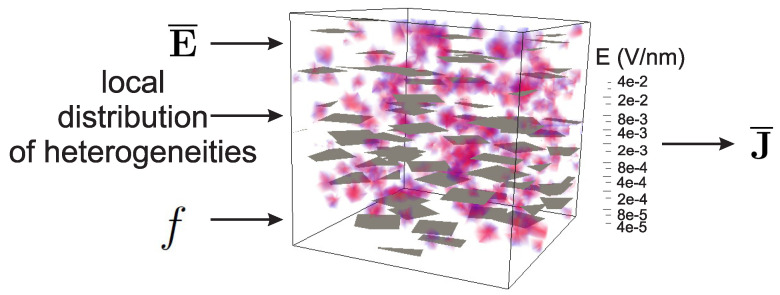
Local model: effective flux $\bar{J}$ depends nonlinearly on the macroscopic electric field $\bar{E}$, volume fraction, and local random distribution of phases.

The objective of the present work was to construct a surrogate model relating macroscopic electric field $\bar{E}$ and volume fraction of graphene inclusions *f* to nonlinear macroscopic electric flux response $\bar{J}$ (see Figure 3). At the microscale, the microstructure was randomly distributed (see Figure 2). Here, due to the scale-separation assumption, it was expected that, despite the random nature of the microstructure, deterministic effective properties at the microscopic scale with respect to the microstructure would be obtained for a given volume fraction.

At the macroscale, uncertainties where then only related to nonhomogeneous distributions of volume fractions. Then, it was assumed that, at the macroscale, the volume fraction was the only stochastic parameter.

### 4.1. Data Generation

We first define a set of *K* electric-field vectors, ${\bar{E}}^{k}={\left\{{\bar{E}}_{1},{\bar{E}}_{2},{\bar{E}}_{3}\right\}}^{k}$, (k=1,2,…,K). The values of ${\bar{E}}^{k}$ were generated using Latin hypercube sampling (LHS) [52]. Then, we define a collection of microstructures as follows. A set of *P* volume fractions are defined, $f^{\alpha }$, α=1,2,…,P. For each volume fraction $f^{\alpha }$, *N* random microstructures satisfying volume fraction $f^{\alpha }$ were generated and are denoted by $\Omega_{\alpha }^{i}$, i=1,2,…,N.

Then, for each macroelectric field vector ${\left\{\bar{E}\right\}}^{k}$, each volume fraction $f^{\alpha }$ and each realization of microstructure $\Omega_{\alpha }^{i}$, nonlinear problem (23) is solved by finite elements to obtain macroelectric displacement vector ${\left\{\bar{J}\right\}}_{\alpha ,i}^{k}$.

As discussed above, the scale-separation assumption allows for removing the stochastic nature of the microstructures at the macroscale. However, due to the RVE size and the random distribution of the inclusions, the outcome intensity of a given electric field is also stochastic. To this purpose, homogenization is performed using stochastic averaging, i.e., for each macroelectric field vector ${\bar{E}}^{k}$ and each volume fraction $f^{\alpha }$, we compute the average over *N* microstructures realizations to obtain ${\bar{J}}_{\alpha }^{k}=\frac{1}{N}\sum_{i=1}^{N}\left({\bar{J}}_{\alpha ,i}^{k}\right)$.

### 4.2. Construction of Neural-Network Surrogate Model

An issue in NN models is that a large set of data may be required to obtain good accuracy, especially for a large number of input parameters [28]. To overcome this limitation, we propose here a hybrid NN/interpolation surrogate model as follows.

First, for each volume fraction $f^{\alpha }$, α=1,2,…,P, used in the training dataset, we define a separate NN, denoted by $N^{\alpha }$, in order to construct the following relationship:

(24) $${\bar{J}}_{\alpha }\left(\bar{E}\right)=N^{\alpha }\left(\bar{E}\right).$$

Then, given $\bar{E}$ and for an arbitrary volume fraction $f\in \left[f^{1},f^{P}\right]$ a Lagrangian interpolation scheme is used to compute $\bar{J}\left(\bar{E},f\right)$ as

(25) $$\bar{J}\left(\bar{E},f\right)=\sum_{j\in N_{k}\left(f\right)}l_{j}\left(f\right){\bar{J}}_{j}\left(\bar{E}\right),$$

where $N_{k}\left(f\right)$ is the set of indices that includes only *k* out of *P* NNs, corresponding to the *k* volume fractions of *f* nearest to those in training dataset $\left\{f^{1},f^{2},\ldots ,f^{P}\right\}$. In Equation (25), $l_{j}\left(f\right)$ are the Lagrangian basis polynomials. Here, k=3 was employed where, as a result, polynomials $l_{j}\left(f\right)$ were second-order.

**Figure 4:**
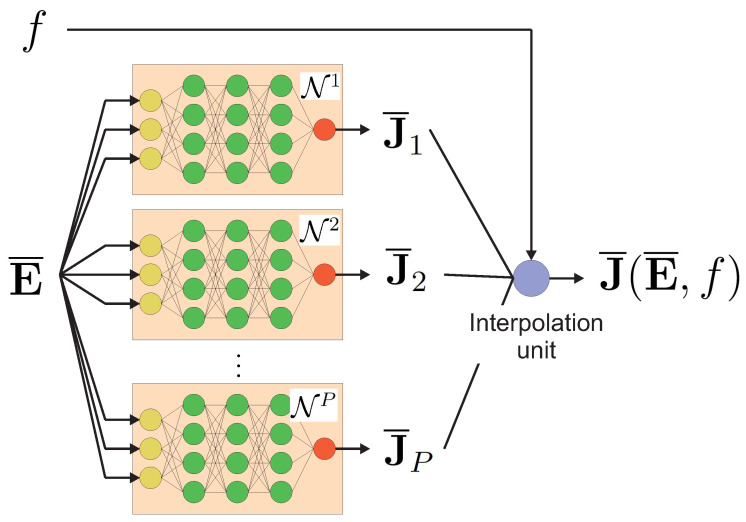
Hybrid neural-network/interpolation surrogate model to describe macroscopic nonlinear behavior.

With this approach, a notion of locality is introduced in the interpolation scheme that leads to better overall predictions, especially in areas where relationship $\left(\bar{E},f\right)-\bar{J}\left(\bar{E},f\right)$ exhibits strong nonlinearity. A schematic of the surrogate-model construction is summarized in Figure 4.

## 5. Nonlinear Stochastic Macroscale Calculations

Here, stochastic macroscopic structural problem is described. At the macroscale, it was assumed that there existed uncertainty in the local volume fraction *f* of the graphene sheets in the general case described by a 3D homogeneous Gaussian stochastic field in the *x*, *y*, and *z* axes. In particular, if x≡(x,y,z), then f(x) is considered to be of the form:

(26) $$f\left(x,\theta \right)=\mu +\sigma f_{0}\left(x,\theta \right).$$

In the above equation, μ and σ are the random field mean value and standard deviation, respectively, θ denotes the random outcome, and $f_{0}\left(x,\theta \right)$ is a zero-mean unit variance Gaussian field with correlation structure $R_{f_{0}}$ given by

(27) $$R_{f_{0}}\left(x_{1},x_{2}\right)=\exp\left[-\left(\frac{|x_{1}-x_{2}|}{{\hat{a}}_{x}}+\frac{|y_{1}-y_{2}|}{{\hat{a}}_{y}}+\frac{|z_{1}-z_{2}|}{{\hat{a}}_{z}}\right)\;\right]$$

where ${\hat{a}}_{x},{\hat{a}}_{y}$ and ${\hat{a}}_{z}$ are correlation length parameters along the *x*, *y*, and *z* axes, respectively.

Next, an approximation of field $f_{0}$ can be obtained using the Karhunen–Loeve series expansion [53]. Specifically, let $\lambda_{n},\phi_{n}$ denote the eigenvalues and eigenfunctions that satisfy the eigenvalue problem $\int R_{f_{0}}\left(x_{1},x_{2}\right)\phi_{n}\left(x_{2}\right)dx_{2}=\lambda_{n}\phi_{n}\left(x_{1}\right)$, ∀n=1,…. This is a Fredholm integral equation and it is typically solved using the finite-element method [53]. Then, $f_{0}$ can be written as

(28) $$f_{0}\left(x,\theta \right)=\sum_{n=1}^{\infty }\sqrt{\lambda_{n}}z_{n}\left(\theta \right)\phi_{n}\left(x\right)$$

with ${\left\{z_{n}\right\}}_{n=1}^{\infty }$ being a series of uncorrelated Gaussian random variables with zero mean and unit variance. In practice, the above series expansion is truncated after $M_{KL}$ terms, giving the following approximation of $f_{0}$.

(29) $$f_{0}\left(x,\theta \right)\approx \sum_{n=1}^{M_{KL}}\sqrt{\lambda_{n}}z_{n}\left(\theta \right)\phi_{n}\left(x\right),$$

which yields, by virtue of Equation (26),

(30) $$f\left(x,\theta \right)\approx \mu +\sigma \sum_{n=1}^{M_{KL}}\sqrt{\lambda_{n}}z_{n}\left(\theta \right)\phi_{n}\left(x\right).$$

Equation (30) allows for us to generate realizations of the field f(x,θ) by generating $M_{KL}$-tuples $\left(z_{1},\ldots ,z_{KL}\right)$ from their distribution. Subsequently, if we consider macrostructure $\bar{\Omega }$ defined in Section 2 and the associated finite-element mesh, at each Gaussian point of element ${\bar{\Omega }}^{e}$, $e=1,2,\ldots ,N_{e}$ with coordinate x, a random value of volume fraction f(x) can be assigned using (30).

During the Newtonian procedure to solve the structural problem, for *f* and $\bar{E}$ given at each Gaussian point of the macromesh structure, the corresponding value of $\bar{J}$ is provided by the surrogate model (25) (see Figure 4). For one realization of the volume-fraction distribution generated by Equation (30), the cost of one two-scale nonlinear structural problem is drastically reduced with the present NN surrogate model, allowing for performing a large number of macrocalculations at a low cost to conduct statistics on quantities of interest in a structure.

**Figure 5:**
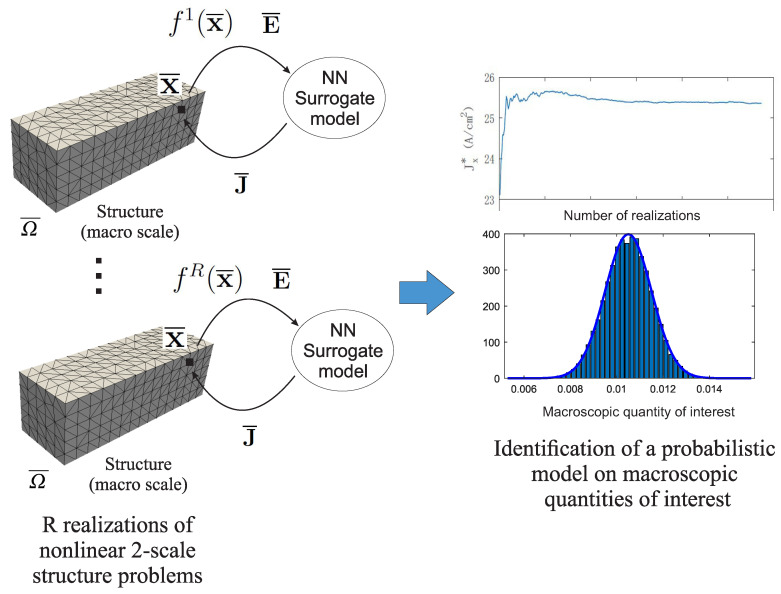
Stochastic nonlinear 2-scale procedure.

Lastly, Monte Carlo simulations were performed on the macroscale problem by evaluating *R* realizations of macrostructures. For each realization r=1,2,…,R, the volume fraction was randomly generated in each Gaussian point by using Equation (30); in total, *R* nonlinear multiscale problems were solved using the above-described procedure. Lastly, statistics can be computed on quantities of interest using the *R* nonlinear FEM solutions. The overall procedure is summarized in Figure 5.

## 6. Numerical Examples

### 6.1. Data Collection

The data were obtained by performing preliminary calculations on the RVE described in Section 4.1. Eight different volume fractions were considered: $f^{1}=0.53\%$, $f^{2}=0.66\%$, $f^{3}=0.79\%$, $f^{4}=0.92\%$,$f^{5}=1.05\%$, $f^{6}=1.19\%$, $f^{7}=1.32\%$, and $f^{8}=1.58\%$ (see Figure 2). For each volume fraction, 15 realizations of random microstructures were generated except for the higher volume fraction, for which only 9 realizations were conducted. For each volume fraction and for each realization, 500 realizations of macroscopic electric field $\bar{E}$ were generated using Latin hypercube sampling. For each case, corresponding electric flux $\bar{J}$ was numerically computed by solving nonlinear Problem (23) on the RVE. The total number of solved nonlinear problems was then 57,000. All these calculations could be performed in parallel.

### 6.2. Validation of Hybrid NN–Interpolation Surrogate Model

The accuracy of the proposed hybrid NN–interpolation surrogate model was first validated by comparing its response with full-field simulations on microstructures for different volume fractions. Regarding the characteristics of the trained neural networks, in all cases, one-hidden-layer architectures were considered with the optimal number of neurons varying for each case, as shown in Table 1. Moreover, the hyperbolic tangent function was employed as the activation function, and Levenberg–Marquardt as the optimizer in all NNs.

**Figure 6:**
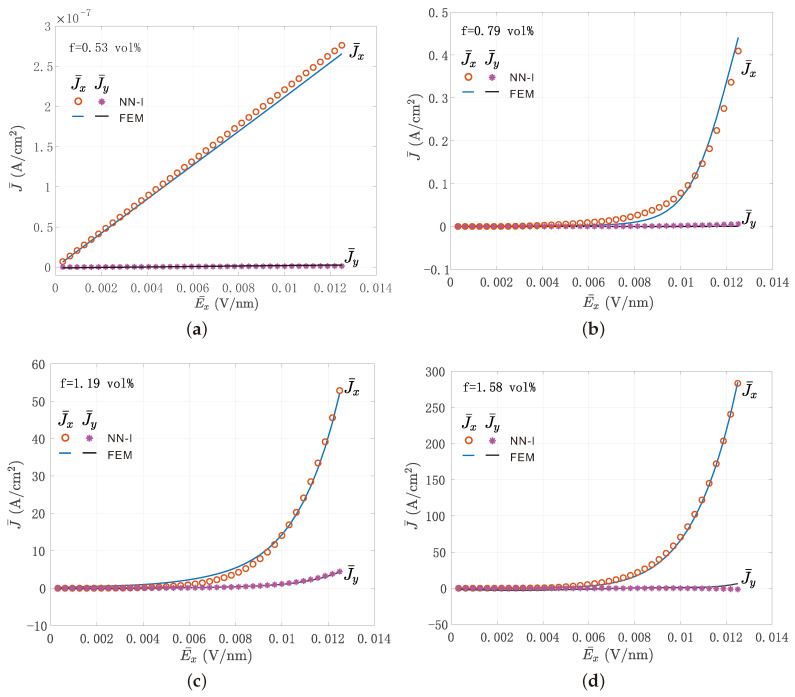
Comparisons between direct simulations obtained by nonlinear FEM calculations on the RVE and the neural-network–interpolation surrogate model: values of ${\bar{J}}_{x}$ as a function of a unidirectional effective electric field ${\bar{E}}_{x}$, ${\bar{E}}_{y}={\bar{E}}_{z}=0$; (**a**) 0.53 vol%, (**b**) 0.79 vol%, (**c**) 1.19 vol%,(**d**) 1.58 vol%.

The plotted curves were obtained as the average over the different realizations of the microstructure. Results are provided in Figure 6. For low volume fractions, the response was linear, while for larger volume fractions, the response was strongly nonlinear. In all cases, the surrogate model could accurately reproduce the effective nonlinear response of the material.

**Figure 7:**
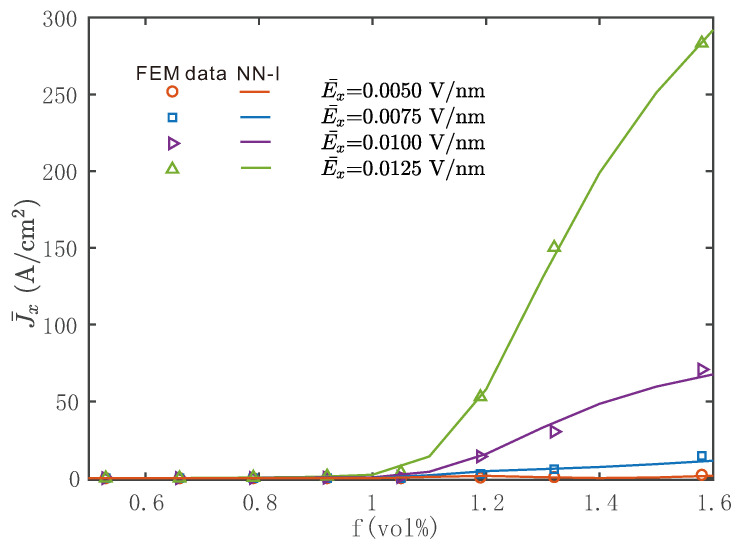
Comparisons between direct simulations obtained by nonlinear FEM calculations on the RVE and NN–I model under various $E_{x}$ ranging from 0.0050 to 0.0125 V/nm: values of $J_{x}$ as a function of the CNT volume fraction, $E_{y}=E_{z}=0$.

A validation of the interpolation procedure described in Section 4.2 is provided in Figure 7, where discrete data obtained by nonlinear FEM calculations on the RVEs are compared to the corresponding model predictions, computed using Equation (25) under various ${\bar{E}}_{x}$ scenarios, with ${\bar{E}}_{y}={\bar{E}}_{z}=0$. The discrete data points obtained by FEM are denoted by marks, while the continuous interpolation with respect to the volume fraction is denoted by solid lines, which confirmed the good accuracy of this scheme.

### 6.3. Stochastic 2-Scale Nonlinear Structure Analysis

**Figure 8:**
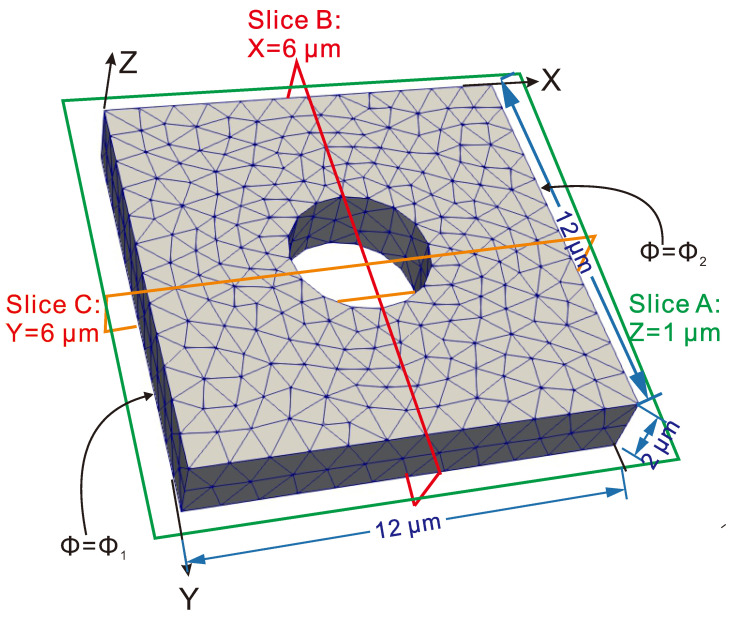
Structural problem: geometry, boundary conditions, and mesh.

In this example, macroscopic stochastic nonlinear computations were performed using the procedure described in Section 5. In particular, 9 different Gaussian fields of volume fractions are investigated at the macroscale, where the studied macrostructure, described in Figure 8, was a plate with a central hole. The plate was subjected to potential boundary conditions such as $\Phi =\Phi_{1}$ on x=0 and $\Phi =\Phi_{2}$ on x=L. A 3D mesh of 1934 elements is used to discretize the domain.

**Figure 9:**
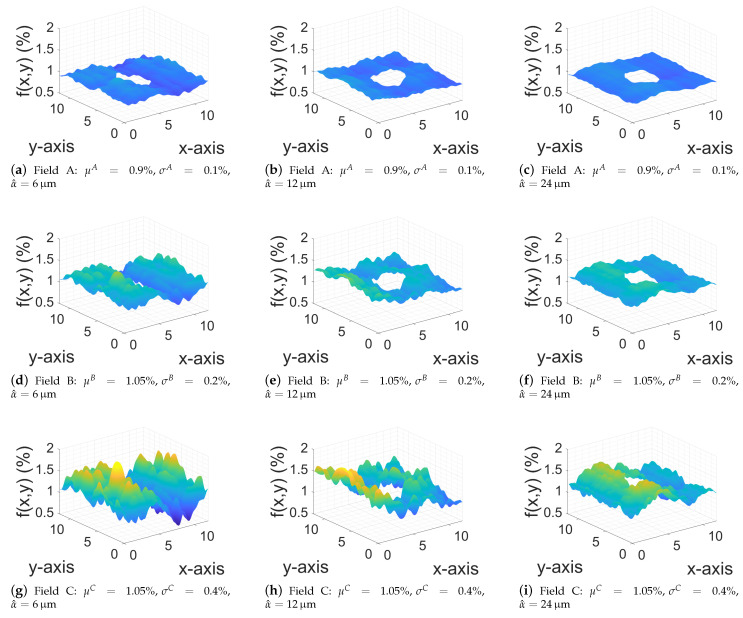
Sample realizations of three Gaussian fields A, B, and C for different correlation lengths $\hat{\alpha }=6,12,24\;\mu$m.

Due to the low thickness of the structure, we assumed that the volume fraction did not vary in the *z* coordinate direction. Next, in order to define the aforementioned Gaussian fields, three different settings were first initialized: for Setting A, we set $\mu^{A}=0.9\%$ and $\sigma^{A}=0.11\;\mu^{A}$; for Setting B, we set $\mu^{B}=1.05\%$ and $\sigma^{B}=0.19\;\mu^{B}$; lastly, for Setting C, $\mu^{C}=1.05\%$ and $\sigma^{C}=0.38\;\mu^{C}$. Then, for each aforementioned setting, we considered ${\hat{a}}_{z}={\hat{a}}_{y}=\hat{a}$ and assign three different values to $\hat{a}$, namely, ${\hat{a}}_{1}=6$ μm, ${\hat{a}}_{2}=12$ μm and ${\hat{a}}_{3}=24$ μm. A sample for each of these fields is illustrated in Figure 9. This figure indicates that an increase in the field standard deviation led to larger variations of volume fraction *f* along the spatial domain. Moreover, a small correlation-length parameter, such as $\hat{a}=6\;\mu$m, produced more “wavy” realizations, while for larger values ($\hat{a}=12$ and 24μm) the realizations became smoother.

**Figure 10:**
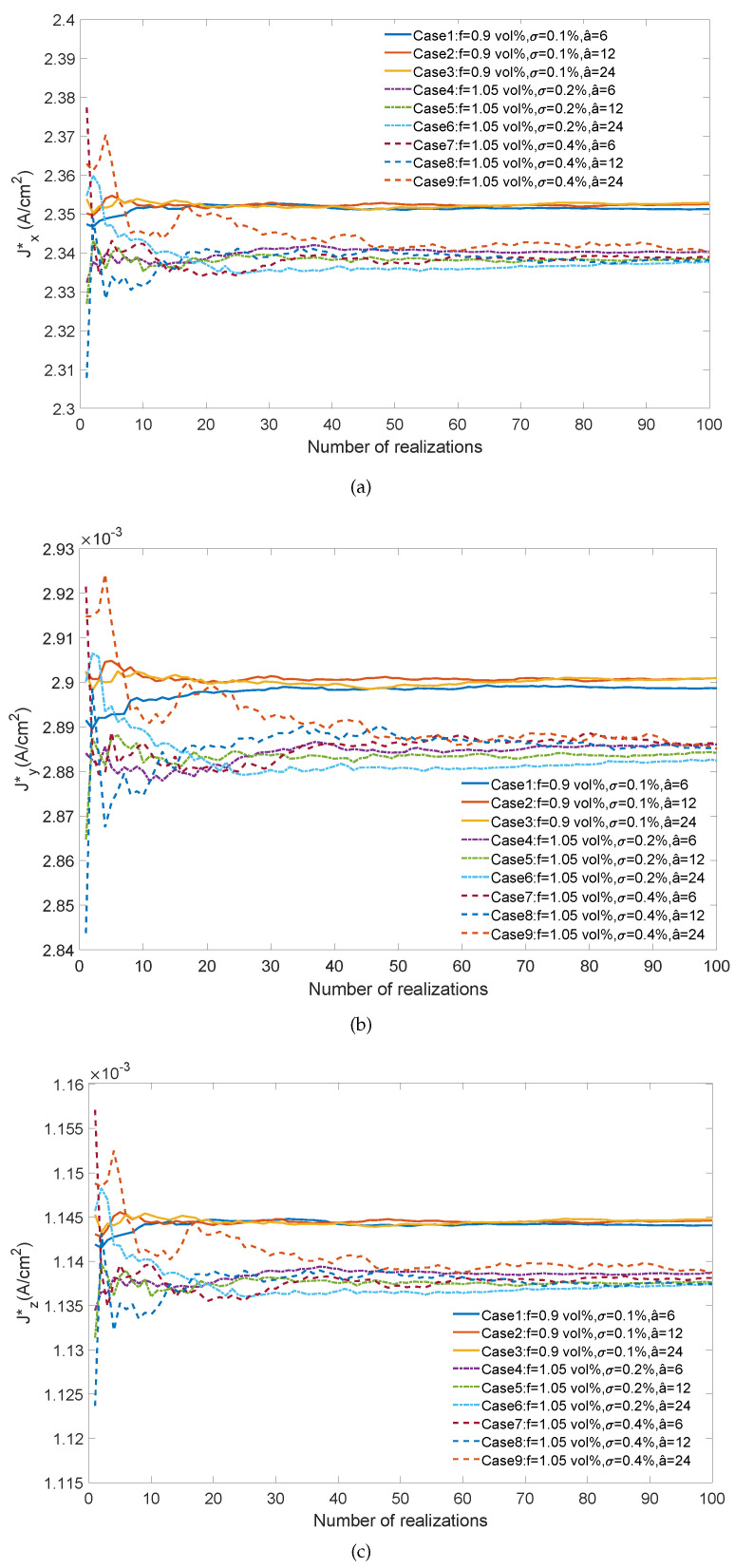
Averaged current-density components as a function of the number of realizations under various distributions of CNT volume fraction and different correlation lengths. (**a**) $J_{x}^{*}$; (**b**) $J_{y}^{*}$; (**c**) $J_{z}^{*}$.

For each of the 3 Gaussian distributions A, B, and C, we analyzed the 3 correlation lengths ${\hat{a}}_{1}$, ${\hat{a}}_{2}$ and ${\hat{a}}_{3}$. For each case, we conducted 100 realizations. Then, in total, we conducted 900 FE${}^{2}$-NN simulations using the procedure described in Section 5. For each one, a stochastic distribution of volume fraction was generated in the elements using (30). The macroscopic quantity of interest is defined here as the average macroscopic flux in the domain $\bar{\Omega }$ as

(31) $$J^{*}=\frac{1}{\bar{V}}\int_{\bar{\Omega }}\bar{J}d\Omega ,$$

where $\bar{V}$ is the volume of $\bar{\Omega }$. The convergence of the components of $J^{*}$ is depicted in Figure 10. In all cases, statistical convergence could be achieved. For the lowest average values *f* and standard deviation σ of the volume fractions (Cases 1–3 in Figure 10), correlation length $\hat{a}$ did not have significant influence on the convergence rate. However, for larger values of *f* and σ, convergence could be much slower (e.g., Case 9 in Figure 10 ), where around 50 realizations are necessary to achieve convergence. This clearly illustrates the interest of the proposed surrogate-based multiscale method, where each realization is performed at the cost of a classical FEM simulation. In contrast, using standard FE${}^{2}$ would not allow performing this kind of statistical analysis with available computer resources.

**Figure 11:**
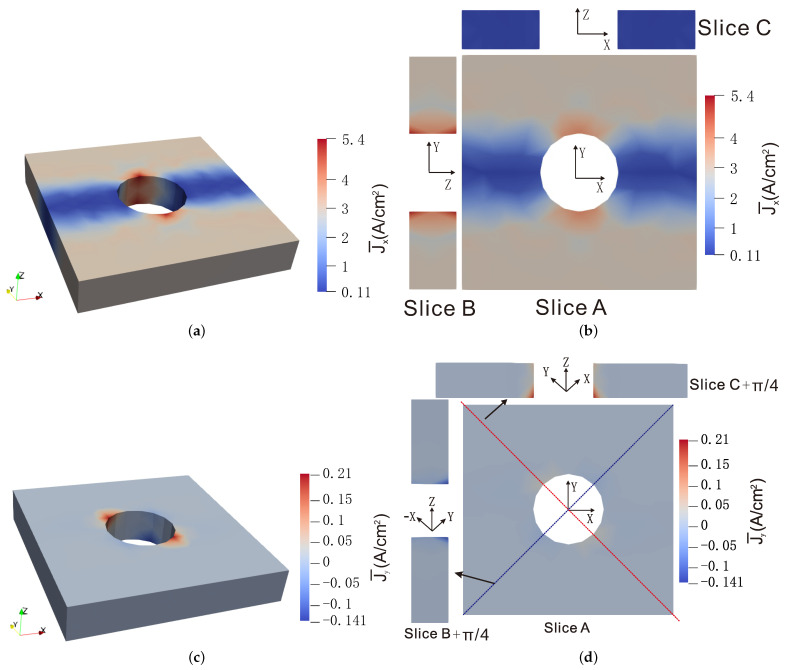
Averaged current density ${\bar{J}}_{x}$ and ${\bar{J}}_{y}$ over 100 realizations calculated by the NN–I model for the composite structure for potential difference $\Phi_{2}-\Phi_{1}=144$ V. The CNT volume fraction obeys distribution A with $\mu^{A}=0.9$ vol%, $\sigma^{A}=0.1\%$, and correlation length $\hat{a}$=6 μm: (**a**) ${\bar{J}}_{x}$-component: 3D view; (**b**) ${\bar{J}}_{x}$-component: plots along different planes; (**c**) ${\bar{J}}_{y}$-component: 3D view; (**d**) ${\bar{J}}_{y}$-component: plots along different planes.

Average distributions of local current densities over 100 realization are plotted in Figure 11 corresponding to distribution A and correlation length $\hat{a}$=6 μm. Clear anisotropy of the effective behavior induced by the aligned graphene sheets along the x−y plane can be appreciated. Comparing Figure 11a,b, we can see a clear difference in the magnitude of the ${\bar{J}}_{x}$ and ${\bar{J}}_{z}$ values, indicating that the effective conductivity in the *z* direction was much lower than that in the x−y plane. The present method could capture such anisotropic behavior in a nonlinear stochastic context.

**Figure 12:**
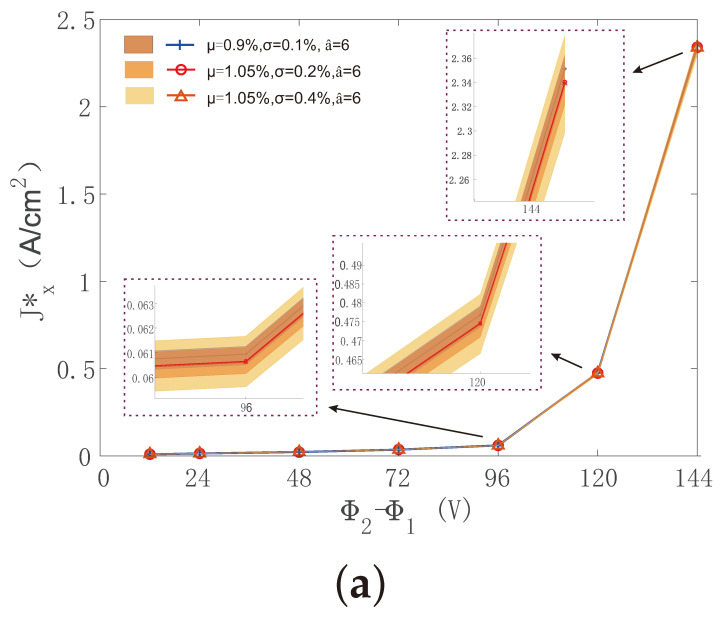

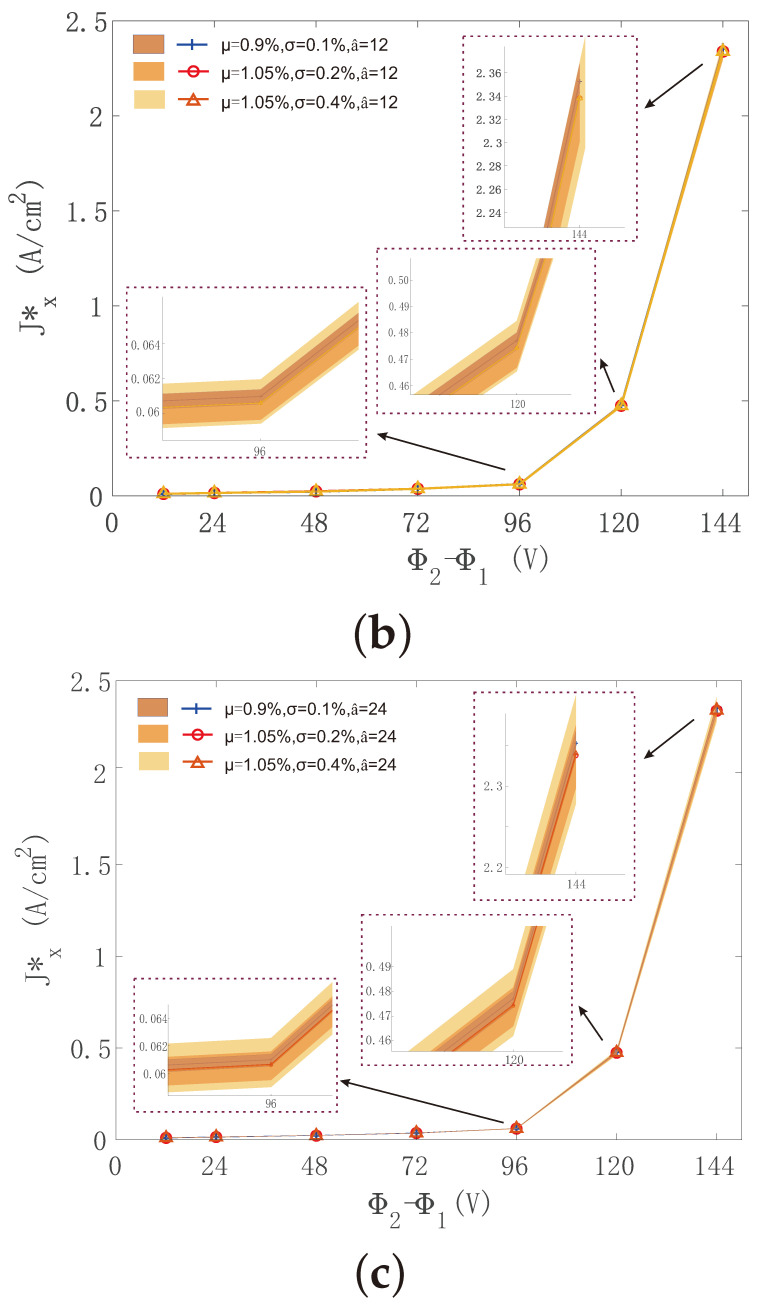
Averaged current density $J_{x}^{*}$ and corresponding deviation as a function of potential difference $\Phi_{2}-\Phi_{1}$ for various distributions of CNT volume fraction under different correlation lengths $\hat{a}$. (**a**) ${\hat{a}}_{1}$ = 6 μm; (**b**) ${\hat{a}}_{2}$ = 12 μm; (**c**) ${\hat{a}}_{3}$ = 24 μm. Color zones indicate ranges of values.

The evolution of the quantity of interest $J_{x}^{*}$ was plotted with respect to the difference of the potential applied over macrostructure $\Phi_{2}-\Phi_{1}$ in Figure 12. Various distributions of CNT volume fractions and different correlation lengths were taken into account for comparison. For each case, 100 realizations were computed, from which we obtained the average and deviation of $J_{x}^{*}$. For instance, in Figure 12a, correlation length $\hat{a}=6$μm is for all three different CNT volume-fraction distributions. The averaged value of $J_{x}^{*}$ was independent on standard deviation σ of the Gaussian distribution, whereas the deviation of $J_{x}^{*}$ increased slightly with increasing σ. The same phenomenon could also be observed in Figure 12b,c. Furthermore, by comparing Figure 12a–c, the increase in correlation length led to a tiny increase in the deviation of $J_{x}^{*}$, but had no effect on its averaged value.

**Figure 13:**
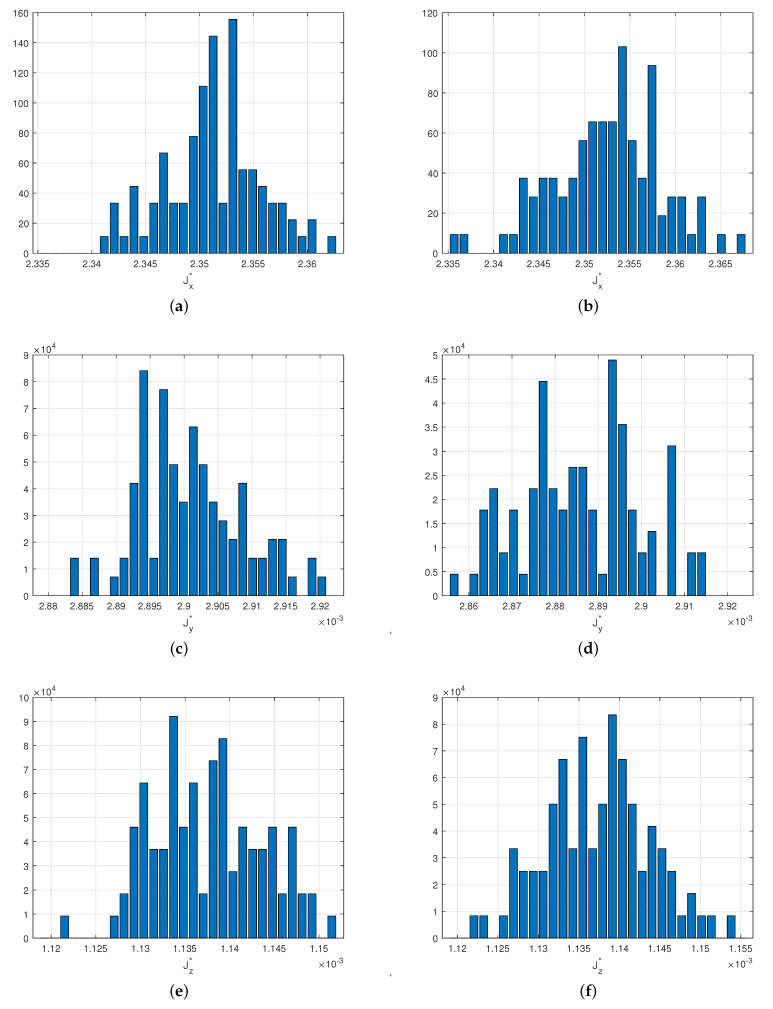
Histograms associated to probabilistic models describing distribution of ${\bar{J}}^{*}$ components at the macroscale. Values of ${\bar{J}}^{*}$ are reported for a fixed value of macroboundary condition $\Phi_{2}-\Phi_{1}=144\;V$; (**a**) $J_{x}^{*}$, μ=0.9%, σ=0.11 μ, $\hat{a}=24$ μm; (**b**) $J_{x}^{*}$, μ=1.05%, σ=0.19 μ, $\hat{a}=24$ μm; (**c**) $J_{y}^{*}$, μ=0.9%, σ=0.11 μ, $\hat{a}=24$ μm; (**d**) $J_{y}^{*}$, μ=1.05%, σ=0.19 μ, $\hat{a}=24$ μm; (**e**) $J_{z}^{*}$, μ=0.9%, σ=0.11 μ, $\hat{a}=24$; (**f**) $J_{z}^{*}$, μ=1.05%, σ=0.19 μ, $\hat{a}=24$ μm.

**Figure 14:**
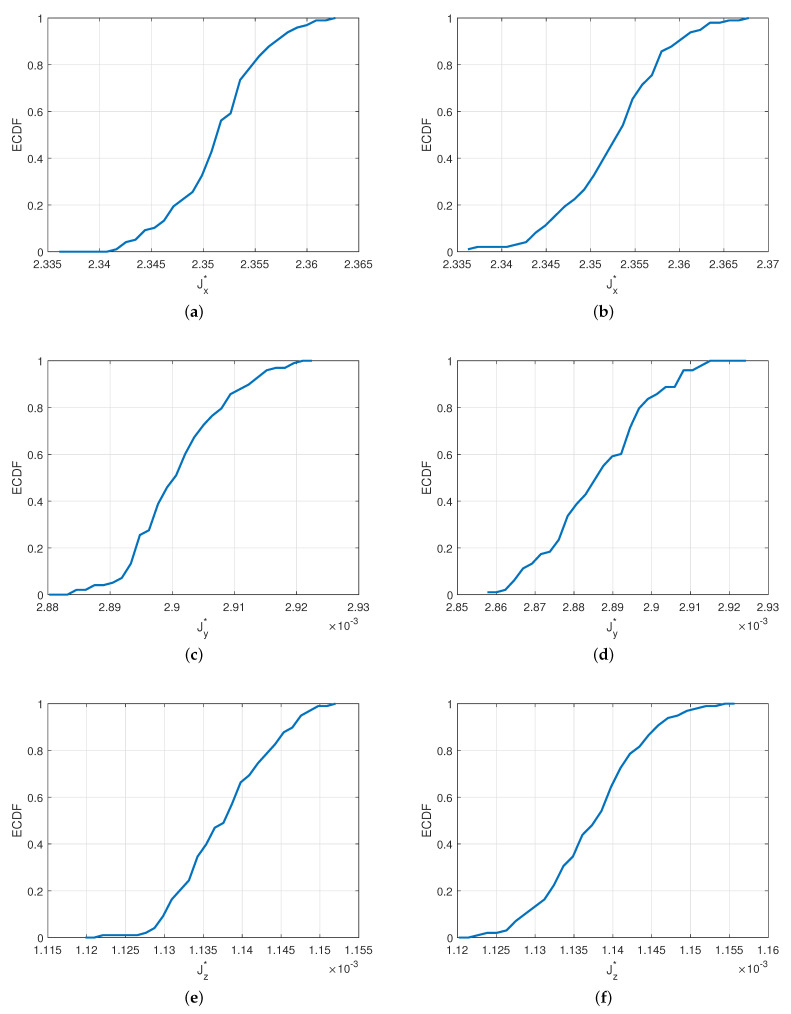
Identified probabilistic models (empirical cumulated distribution functions) for generating distributions of ${\bar{J}}^{*}$ components at the macroscale. Values of ${\bar{J}}^{*}$ are reported for a fixed value of macroboundary condition $\Phi_{2}-\Phi_{1}=144$ V; (**a**) $J_{x}^{*}$, μ=0.9%, σ=0.11 μ, $\hat{a}=24$ μm; (**b**) $J_{x}^{*}$, μ=1.05%, σ=0.19 μ, $\hat{a}=24$ μm; (**c**) $J_{y}^{*}$, μ=0.9%, σ=0.11 μ, $\hat{a}=24$ μm; (**d**) $J_{y}^{*}$, μ=1.05%, σ=0.19 μ, $\hat{a}=24$ μm; (**e**) $J_{z}^{*}$, μ=0.9%, σ=0.11 μ, $\hat{a}=24$; (**f**) $J_{z}^{*}$, μ=1.05%, σ=0.19 μ, $\hat{a}=24$ μm.

Lastly, in Figure 13, distributions of target values $J_{x}^{*}$, $J_{y}^{*}$ and $J_{z}^{*}$ are plotted for selected cases of the probabilistic models describing the distribution of the volume fraction in the macroscale. In Figure 14, the associated empirical cumulative distribution functions (ECDFs) are provided. These functions were identified from the histograms in Figure 13. These allow for a direct quantitative reading of key values of interest (minimum, maximum, mean, percentiles, etc.) regarding the macroscopic quantities. ECDFs also have the property of converging to the true CDF of the stochastic quantities of interest as the number of samples is increased [54]. Typically, an accurate estimate of a CDF would require a very large number of samples (>10${}^{5}$); however, performing these many evaluations of nonlinear multiscale models would be computationally prohibitive. In this regard, the use of the proposed surrogate is the only viable approach to obtain reliable approximations of the CDFs under investigation. This demonstrates the potential of the present approach in constructing probabilistic models for macroquantities of interest in nonlinear multiscale models of random materials.

## 7. Conclusions

A stochastic data-driven multilevel finite-element (FE${}^{2}$) method was proposed to solve nonlinear heterogeneous structures with uncertainties at both the micro- and the macrolevel. A hybrid neural-network–interpolation (NN–I) scheme was developed to improve the accuracy of NN surrogate models, allowing for the use of a lower number of representative volume element (RVE) nonlinear calculations, which serve as a database to train the neural networks. This NN–I surrogate model was used to develop a data-driven method for nonlinear heterogeneous conduction in a stochastic framework: uncertainties can be included on both the micro- and the macrolevel. More specifically, the drastic reduction in computational costs brought by the NN-I surrogate model allows Monte Carlo simulations of nonlinear heterogeneous structures. This framework was applied to propagate uncertainties in such nonlinear multiscale models, and demonstrated that it can be used to identify probabilistic models related to some quantities of interest at the macroscale in a fully nonlinear, anisotropic context.

## Figures and Tables

**Table 1:** Characteristics of neural networks.

Case (Vol%)	Number of Neurons	MSE (Validation Set)
0.53	16	1.167 $\times 10^{-18}$
0.66	23	6.785 $\times 10^{-18}$
0.79	7	4.006 $\times 10^{-12}$
0.92	18	8.941 $\times 10^{-7}$
1.05	77	1.579 $\times 10^{-5}$
1.19	59	3.465 $\times 10^{-4}$
1.32	36	1.243 $\times 10^{-1}$
1.58	74	4.790 $\times 10^{-2}$

## Data Availability

The data that support the findings of this study are available from the corresponding author upon reasonable request.
